# Diagnosis System of Microscopic Hyperspectral Image of Hepatobiliary Tumors Based on Convolutional Neural Network

**DOI:** 10.1155/2022/3794844

**Published:** 2022-03-17

**Authors:** Shixin Huang, Jiawei Luo, Kexue Pu, Min Wu

**Affiliations:** ^1^School of Communication and Information Engineering, Chongqing University of Posts and Telecommunications, Nanan 400065, Chongqing, China; ^2^Department of Scientific Research, The People's Hospital of Yubei District of Chongqing City, Yubei 401120, Chongqing, China; ^3^West China Biomedical Big Data Center, West China School of Medicine, Sichuan University, Chengdu 610041, Sichuan, China; ^4^School of Medical Informatics, Chongqing Medical University, Yuzhong 400016, Chongqing, China; ^5^Department of Radiology, The People's Hospital of Yubei District of Chongqing City, Yubei 401120, Chongqing, China

## Abstract

Hepatobiliary tumor is one of the common tumors and cancers in medicine, which seriously affects people's lives, so how to accurately diagnose it is a very serious problem. This article mainly studies a diagnostic method of microscopic images of liver and gallbladder tumors. Under this research direction, this article proposes to use convolutional neural network to learn and use hyperspectral images to diagnose it. It is found that the addition of the convolutional neural network can greatly improve the actual map classification and the accuracy of the map, and effectively improve the success rate of the treatment. At the same time, the article designs related experiments to compare its feature extraction performance and classification situation. The experimental results in this article show that the improved diagnostic method based on convolutional neural network has an accuracy rate of 85%–90%, which is as high as 6%–8% compared with the traditional accuracy rate, and thus it effectively improves the clinical problem of hepatobiliary tumor treatment.

## 1. Introduction

Different from regular color images and grayscale images, in addition to spatial structure information, hyperspectral images also contain spectral information, so it will definitely increase the multisource information of hyperspectral images. Hyperspectral images can be used to identify more complex purposes based on the multisource characteristics of information. However, the rich spectral information can easily cause data complexity and affect classification performance. The development of deep learning theory, especially the development of convolutional neural networks, provides effective tools for hyperspectral remote sensing images in medical diagnosis.

Feature extraction and classification technology based on hyperspectral data has always become one of the hot topics in the field of remote sensing. The existing feature extraction methods use linear or nonlinear equations, mainly focusing on specific side features of ground features. To manually design or specify the extracted features, the process of manually selecting features requires professional knowledge and experience, and it takes a lot of time. Using convolutional neural networks, the computer can automatically learn functions that are beneficial to the task. Combining this process with a part of model training can further improve the accuracy of classification and recognition. To apply convolutional neural networks to images, Jaderberg et al. proposed an end-to-end text recognition system—locating and recognizing text in natural scene images—and text-based image retrieval. The system is based on a region proposal mechanism for detection and a deep convolutional neural network for recognition [1]. The focus of his research is on text recognition, although there are applications for image retrieval, they are not very useful. The large difference between natural images and medical images based on deep learning may oppose this kind of knowledge transfer. Tajbakhsh et al. consider four different medical imaging applications in three majors (radiology, cardiology, and gastroenterology), which involve classification, detection, and segmentation from three different imaging modes. He also studied the performance of deep CNN trained from scratch and fine-tuned pretrained CNN in a hierarchical manner [2]. He applies deep neural networks in the medical field, but it involves radiology, cardiology, and gastroenterology. This article mainly studies the application of convolutional neural network microscopic hyperspectral images in the diagnosis of hepatobiliary tumors. Among brain tumors, glioma is the most common and aggressive, resulting in a very short life expectancy at its highest level. Magnetic resonance imaging (MRI) is an imaging technique widely used to evaluate these tumors, but the large amount of data generated by MRI prevents manual segmentation in a reasonable time and limits the use of precise quantitative measurements in clinical practice. Pereira et al. proposed an automatic segmentation method based on convolutional neural network (CNN) to explore small 3 × 3 kernels [3]. He applied the CNN to segmentation exploration to diagnose brain tumors, which is worth studying in this article. If it can be applied to the diagnosis of liver and gallbladder tumors, it will be closer to the subject of this article. Hepatectomy is the gold standard curative treatment for liver tumors in patients with preserved liver function. Many large tumors require extended hepatectomy (EH), and Attili et al. goal is to describe his EH results for large hepatobiliary tumors [4]. He studied the experimental results of hepatectomy, but this article mainly studies the diagnosis methods of hepatobiliary tumors. To understand the relevant research related to hepatocellular carcinoma, the purpose of Kamil and Rowe is to illustrate the favorable and unfavorable results of MRI results in predicting the prognosis of hepatocellular carcinoma (HCC). He also discussed the clinical significance of HCC MR results and correlated them with biological behavior [5]. His research is to predict the clinical significance of HCC and HCC MR. This article can refer to its diagnostic methods in predicting HCC. The purpose of Jiang et al. study is to use a multi-omics platform to determine the clonal origin and metastasis relationship of simultaneous multifocal tumors in the hepatobiliary and pancreatic systems [6]. His research is the diagnosis method of simultaneous multifocal tumors, and this article mainly studies the diagnosis method of hepatobiliary tumors with microscopic hyperspectral images. Human telomerase reverse transcriptase (h-TERt) and its components play an important role in cancer progression, but recent data indicate that changes in telomerase and telomerase can be found in other diseases; increasing evidence shows that this enzyme plays a key role in the field of hepatobiliary and pancreatic diseases. Vito reviewed related publications analyzing the correlation between telomerase activity and hepatobiliary and pancreatic diseases. The results indicate that telomerase reactivation plays an important role in the occurrence and progression of hepatobiliary and pancreatic tumors, and can be used as a diagnostic biomarker, prognostic predictor, and a promising therapeutic target for hepatobiliary and pancreatic cancer [7]. His research is the treatment of hepatobiliary and pancreatic diseases. Although it is not related to the diagnosis of hepatobiliary tumors in this article, it still has a certain reference value. The abovementioned documents are quite good in the structure of the article and the grasp of related technologies, but most of them are based on experiments under ideal conditions. The exploration of the practical feasibility of the experiment is still not enough.

The innovation of this paper is using hyperspectral image imaging features and the technical support of hyperspectral image technology and CNNs as the theoretical basis, the original CNN method is improved, and a new deep learning method is proposed. The accuracy of the hyperspectral image is improved through learning, and the performance of the hyperspectral image in diagnosing hepatobiliary tumors is improved through the SDCNN model.

## 2. Hyperspectral Image Diagnosis Method

### 2.1. Overview of Hyperspectral Image Technology

**Figure 1:**
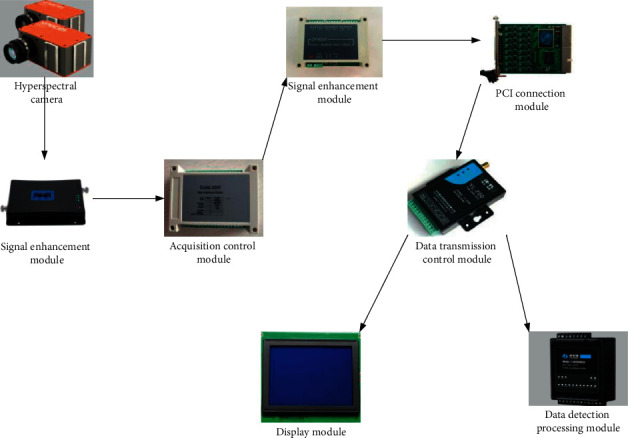
Hyperspectral image acquisition process.

Hyperspectral imaging [8], refers to the combination of image and spectroscopic techniques to obtain 3D cube data of hyperspectral images [9]. Hyperspectral cube images do not actually represent spatial 3D images [10]. Strictly speaking, the hyperspectral image should be a 2.5D image data type. In terms of images, most of the digital pictures that people usually see are RGB images, which are composed of three basic colors red, green, and blue. In other words, an RGB image can be split into the red, green, and blue components. Each component can generate a grayscale image of the image (what people call a black-and-white image). In a digital image, this grayscale image is composed of a 2D data matrix, and each data in the matrix are a so-called pixel. Such as a 56 × 56 RGB image, the actual size of its data storage is 56 × 56 × 3, and 3 represents its three RGB components. If these 3 components are extended to contiguous hundreds or thousands of bands, such as 100 continuous bands, the image data will be expanded to 56 × 56 × 100. And this 100 is the expansion of the spectrum, which adds rich spectrum information to the image. As shown in Figure 1, it is the image acquisition process of hyperspectral imaging technology.

**Figure 2:**
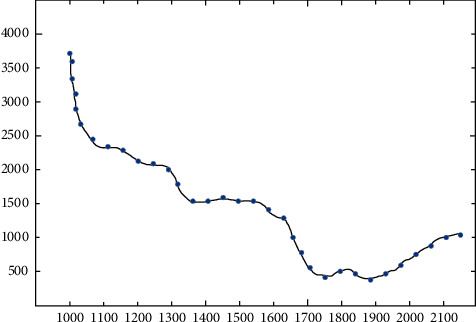
The spectrum of a point on the hyperspectral image.

As shown in Figure 2, when the *x* and *y* of a hyperspectral image represent its image in the pixel dimension, *λ* is the spectral dimension [11]. If you take a point from the image dimension, this point can be connected in the spectral dimension to get the spectrum of this point.

**Figure 3:**
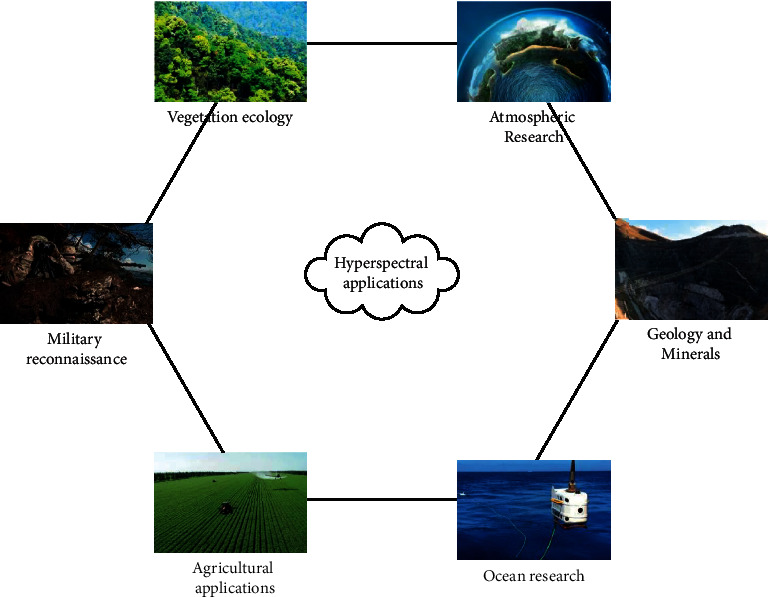
Application of hyperspectral image acquisition.

Hyperspectral image acquisition equipment can usually provide continuous band images from visible light to near-infrared region, and these band images have higher discreteness than RGB images [12], and the band interval is also narrower. As hyperspectral images provide rich information in spatial and spectral dimensions and the resolution is high, it was first applied to remote sensing analysis and later applied to medicine, chemistry, precision agriculture, and environmental engineering. Its application is shown in Figure 3:

For hyperspectral images, its imaging process can be divided into two main techniques: spectroscopy [13] and filter method [14]. Among them, the spectroscopy can be subdivided into prism spectroscopy, grating spectroscopy, interference spectroscopy, etc., according to the implementation technology. Spectroscopy usually scans the spatial dimension to obtain the spectral curve of a row of pixels in the space. The filter method can be subdivided into acousto-optic tunable filters, coated filters, liquid crystal filters, etc. according to the implementation technology. The filter method usually scans the spectrum dimension to obtain images of different wavelengths. To obtain the spectrum and images at the same time, multi-CCD imaging technology and two-dimensional grating imaging technology can be used.

There are usually three mainstream storage formats for hyperspectral data: bands are interleaved by pixels, bands are interleaved by rows and in the order of bands. The BIP storage method stores pixel data in the order of pixels, with the pixel as the main body, and the current pixel values in all bands are continuously put together, to provide the best performance for the spectral information access of the image, but this method breaks the spatial continuity of different pixels. In the BSQ method [15], when storing, each row of data immediately follows the next row in the same band. That is, all pixels of the same waveband are arranged together, and all pixels of different wavebands are directly stored in sequence. This storage method can quickly access any spatial position of a single band when performing spatial analysis on the image, and its spatial processing ability is relatively strong. However, this kind of access method will take a lot of time when it needs to perform spectral analysis on hyperspectral data with a large number of bands and large image data. BIL [16] is a performance compromise between BIP and BSQ; specifically, it treats the upper row of the space as a whole. This new small whole is arranged in the order of bands, to achieve a compromise between spatial and spectral information access speed. In MATLAB, the multiband binary hyperspectral image data in the specified format can be read through the multibandread function. At the same time, the image array can be saved as a binary image through the multibandwrite function (the image format can be defined as BIP, BIL, or BSQ in the function). When using the hyperspectral image professional processing software ENVI to store data, the internal read and write mode of the data by setting the storage format to BIP, BIL, or BSQ can be designed. Different from other images, hyperspectral images have their particularities, including large amount of data, complex preprocessing algorithms, etc., which create the complexity and professionalism of the hyperspectral image processing system. Hyperspectral images are stored in the form of image three-dimensional cubes [17]. From the perspective of data structure, its salient features mainly include three aspects. First of all, there are a large number of hyperspectral image bands, often containing dozens or hundreds of bands, and each band may be used alone for the analysis of certain applications. For example, the spectral curve of plants is often between the visible light waveband and the near-infrared waveband, about 0.76 microns, and the reflectance rises sharply. The absorption of electromagnetic waves in the 0.4–2.5 micron range by relatively pure natural surface water is significantly higher than that of most other ground objects. Second, the hyperspectral image combines the highly variable spectral information with the spatial dimension information of the image. This makes the image contain rich spatial and spectral information of the ground objects at the same time. This information shows the characteristics of the spatial distribution of ground objects. At the same time, one of the pixels can be used to obtain its spectral characteristics: the spectrum is unified. Finally, any band of hyperspectral images can only absorb photons in the current band for imaging, so the spatial resolution is low.

### 2.2. Convolutional Neural Network

In the hyperspectral image classification problem based on CNNs, the commonly used CNN models can be roughly divided into two types. One is that the convolution kernel and model parameters are iteratively updated through error feedback, for example, traditional CNNs or variants of CNNs such as AlexNet; the other is a CNN that the convolution kernel learns in advance. The characteristic of this type of network is that the convolution kernel is prelearned by related algorithms. The error back propagation only updates the fully connected network and its classifier parameters. In the CNN model of the abovementioned prelearned convolution kernel, the PCA algorithm [18], the Random algorithm [19,20], and the K-means algorithm [21] are prelearned to generate the convolution kernel, and the trained convolution kernel is obtained. The network model then performs the task of hyperspectral image classification. For the current CNN model, especially the CNN model that learns the convolution kernel in advance, the number of convolution kernels is often set by artificial experience, which is often inconsistent with the problem. And it has a negative effect on the network classification effect, which reduces the classification accuracy. Therefore, this chapter proposes a CNN classification method based on the adaptive decision of the number of convolution kernels [22], and applies it to the task of hyperspectral image classification. This method uses a clustering method to quickly find and obtain the density peak used to determine the number of convolution kernels. This article proposes an adaptive method based on clustering to determine the number of convolution kernels. In order to solve the problem of incorrect convolution kernel decision in the clustering-based adaptive decision method for the number of convolution kernels, this chapter also proposes a joint algorithm based on the similarity and density of image blocks based on the CFSFDP method. The metric generation algorithm of the convolution kernel (herein referred to as the MCSFDP method) effectively improves the accuracy of the convolution kernel and further improves the accuracy of the hyperspectral image.

The core idea of CFSFDP clustering algorithm lies in the characterization of cluster centers. First, the cluster center point is surrounded by adjacent data points whose density is less than this point. Second, the density of cluster center points is greater than the density of other data points. Based on this core idea, CFSFDP uses two variables, local density and distance, to characterize the mathematical properties of data points, as follows:

The assumption here satisfies the triangle inequality. The local density *ρ*_*i*_ of data point *i* is defined as shown in the following equation (1):

(1) $$\begin{array}{c} \rho_{i}=\sum_{j}X\left(d_{ij}-d_{c}\right). \end{array}$$

The algorithm is only sensitive to the relative size of different data point densities *ρ*_*i*_ . For data sets with a large number of samples, the analysis results are robust and depend on the choice of the cutoff distance *d*_*c*_. The judgment of *δ*_*i*_ is obtained by calculating the minimum distance between data point *i* and other data points with greater density, as shown in the following equation (2):

(2) $$\begin{array}{c} \delta_{i}=\underset{j:\rho_{j}>\rho_{i}}{\min}\left(d_{ij}\right). \end{array}$$

For the data point with the largest density value, its distance is the maximum value of the distance between the data point and all other points, which is expressed as follows:

(3) $$\begin{array}{c} \delta_{i}=\max_{j}\left(d_{ij}\right). \end{array}$$

**Figure 4:**
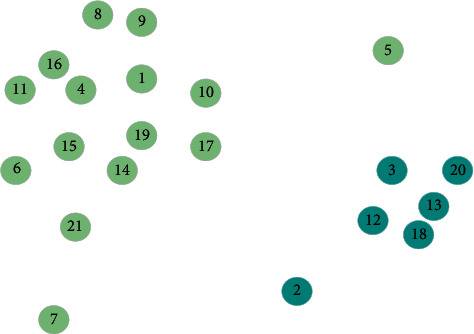
Distribution of data points.

Figure 4 shows a data set with 21 two-dimensional data points.

**Figure 5:**
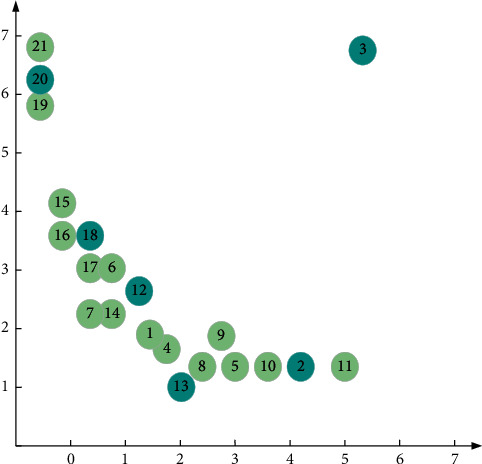
Decision diagram of data points on the density and distance plane.

Figure 5 is a decision diagram for the data points in Figure 4 on the distance and density planes. As shown in Figure 5, the 1st and 10th data points have larger *ρ* and *δ* values at the same time, and the 1st and 10th data points happen to be the two cluster centers of the data set. Therefore, the cluster center point is defined as the data point with larger *ρ* and *δ* values at the same time.

For data set projection decision graphs that cannot be distinguished by the naked eye, the CFSFDP clustering algorithm introduces a comprehensive consideration value *γ*_*i*_ based on the data point density *ρ*_*i*_ and distance *δ*_*i*_, as shown in the following formula (4):

(4) $$\begin{array}{c} \gamma_{i}=\rho_{i}\delta_{i}. \end{array}$$

In this section, based on the adaptive decision of the number of convolution kernels, the focus is on the hyperspectral image CNN classification method proposed in this article. Different from the traditional CNN model, the convolution kernel is updated during the network training process. This section uses the CNN model based on the prelearned convolution kernel. The network structure uses a single convolutional layer of CNN structure. It includes an input layer, a convolutional layer, a pooling layer, a fully connected layer, and a classification layer.

The basic idea of the hyperspectral image CNN classification method based on adaptive decision of the number of convolution kernels is as follows: First, a method based on clustering to adaptively determine the number of convolution kernels is proposed. Automatically, the number of sub-image blocks, which is suitable for determining the number of convolution kernels, is determined. That is to say, according to CFSFDP [23] to determine the adaptation method of the number of convolution kernels. Next, it proposes a convolutional CFSFDP method based on the joint measurement of image block similarity and density. The kernel generation algorithm refers to only the distance similarity threshold, which is used to replace the common limit value of distance and density of CFSFDP algorithm. Finally, the adaptively determined number of convolution kernels generated by the MCFSFDP algorithm is used for hyperspectral image classification. The network model of the adaptively determined CNN based on the number of convolution kernels is called the MCSFDP-based CNN.

**Figure 6:**
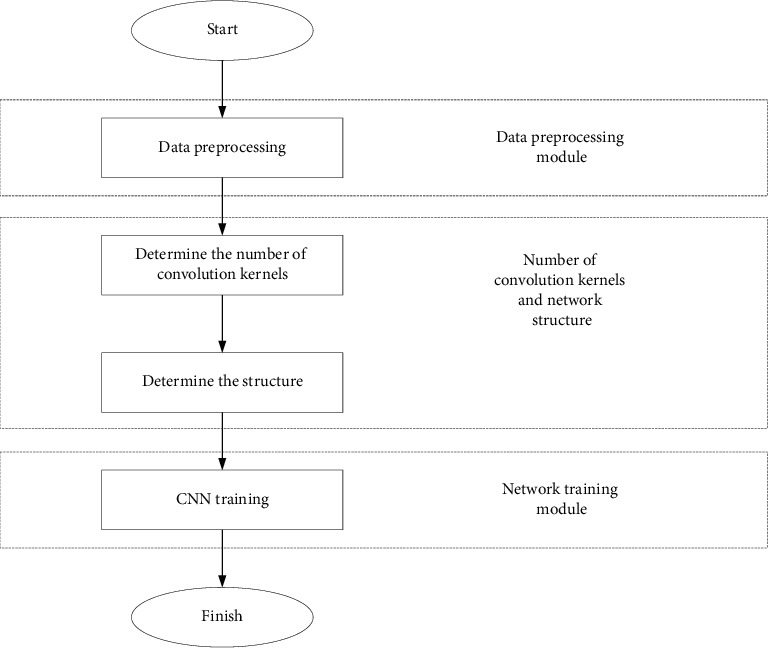
Schematic diagram of convolutional neural network hyperspectral image classification algorithm.

The CNN based on MCFSFDP includes the following three main modules: (1) Data preprocessing module, that is the training, verification, and test sample image blocks extracted from the hyperspectral image and sub-image block samples required for clustering; (2) The MCFSFDP method clusters to generate a convolution kernel module, and the MCFSFDP clusters sub-image block samples extracted from the training sample image block to generate a CNN convolution kernel, and at the same time obtains a CNN model; (3) CNN model training and hyperspectral image classification module. The schematic diagram of the proposed method is shown in Figure 6:

In the CNN model based on the prelearned convolution kernel, the extracted subpatch samples are used. Pre-generating convolution kernels and adaptively determining the number of convolution kernels, it is necessary to choose an appropriate clustering method to achieve this goal. Therefore, a variety of clustering methods have been proposed. Among these clustering algorithms, the clustering method that quickly finds and obtains the density peak is a representative method, which can automatically determine the number of cluster centers. The main advantage of CFSFDP clustering algorithm comes from its core idea, “The cluster center has a higher density than its neighboring points, and has a relatively large distance from the data points with greater density,” that is the cluster center point is determined by two thresholds, namely density and distance.

The calculation of the relative amount of two data points is as follows:

(5) $$\begin{array}{c} \rho_{j}=\sum_{j}X\left(d_{jk}-d_{c}\right), \end{array}$$

when:

(6) $$\begin{array}{c} d_{jk}-d_{c}<0, \end{array}$$

(7) $$\begin{array}{c} X\left(d_{jk}-d_{c}\right)=1, \end{array}$$

otherwise:

(8) $$\begin{array}{c} X\left(d_{jk}-d_{c}\right)=0. \end{array}$$

Distance between data points:

(9) $$\begin{array}{c} \delta_{j}=\underset{k}{\min}\left(d_{jk}\right). \end{array}$$

For the point *j* with the maximum density, its distance is the maximum distance from all other points, expressed as:

(10) $$\begin{array}{c} \delta_{j}=\max_{k}\left(d_{jk}\right). \end{array}$$

To adaptively obtain the convolution kernel and select the number of convolution kernels, following calculation steps to obtain the optimal distance threshold *δ*_*A*_ are followed:

(11) $$\begin{array}{c} {\text{num}}_{v}=f\left(\delta_{v}\right). \end{array}$$

The calculation of the number of sample data points for the sub-image block is as follows:

(12) $$\begin{array}{c} {\text{con}}_{v}=\frac{\left[f\left(\delta_{v+1}\right)-f\left(\delta_{v}\right)\right]}{\left(\delta_{v+1}-\delta_{v}\right)}. \end{array}$$

The absolute value of the result can be obtained:

(13) $$\begin{array}{c} g_{ij}^{k}=\max\left(f_{1+p\left(i-1\right)}^{k},f_{pi,1+p\left(j-1\right)}^{k}\right){\text{quo}}_{v}=\left|\frac{{\text{con}}_{v}}{{\text{con}}_{v+1}}\right|. \end{array}$$

The feature image after *k* times of convolution kernel convolution is as follows:

(14) $$\begin{array}{c} f\in R^{\left(m-n+1\right)\times \left(m-n+1\right)}\\ f_{ij}^{k}=\sigma \left(\sum_{c}\sum_{a=0}^{n-1}\sum_{b=0}^{n-1}w_{\text{abc}}^{k}x_{i+a,j+b}^{c}\right). \end{array}$$

The *k*-th feature map obtained after convolution is the result of downsampling. The sampling operation is a nonspatial overlapping sampling method, which is calculated by the following formula (15):

(15) $$\begin{array}{c} \max=f_{1+p\left(i+1\right)}^{k}+f_{pi}^{k}\\ g\in R^{\left(m-n+1\right)\left(m-n+1\right)/p}, \end{array}$$

(16) $$\begin{array}{c} \max=f_{1+p\left(i+1\right)}^{k}+f_{pi}^{k}\\ g\in R^{\left(m-n+1\right)\left(m-n+1\right)/p}, \end{array}$$

where *P* is the local size of the space,

(17) $$\begin{array}{c} j\leq \frac{\left(m-n+1\right)}{p}. \end{array}$$

Finally, input the test sample image block into the trained CNN to obtain the hyperspectral image classification test results. The evaluation indicators for the existing clustering results are as follows:

Rand Index:

(18) $$\begin{array}{c} \text{RI}=\frac{a+b}{C_{2}^{n}}, \end{array}$$

where *C*_2_^*n*^ is the total number of pairs of sample elements that can be formed in the data set. The larger the Rand index value RI, the more consistent the clustering result is with the real situation of the sample element category.

Standard mutual information:

(19) $$\begin{array}{c} I\left(A,B\right)\log_{2}\frac{P\left(AB\right)}{P\left(A\right)P\left(B\right)},\\ =\log_{2}\frac{P\left(A|B\right)}{P\left(A\right)},\\ =\log_{2}\frac{P\left(B|A\right)}{P\left(B\right)}. \end{array}$$

The size and method of the convolution kernel are adaptively determined based on the weighted distance measurement. At the same time, the convolution kernel is used for CNN classification of hyperspectral images. This is to fold the size of the kernel to adapt to the image CNN classification method.

### 2.3. Imaging Characteristics of Hyperspectral Remote Sensing

The analysis of the characteristics of hyperspectral data is the study of the feature extraction and classification methods of hyperspectral images, especially the basis of the analysis of signs and statistical characteristics of hyperspectral data, and the processing and analysis methods of hyperspectral data. This part mainly introduces the imaging characteristics and data performance of hyperspectral images. On this basis, the statistical characteristics of image space, spectral space, and feature space are analyzed to show the effectiveness of spatial spectral information combination in hyperspectral classification.

Compared with ordinary remote sensors, imaging spectrometers have more spectral channels and narrower band widths within a certain wavelength range. There is spectral overlap between the bands, so the acquired remote sensing images have a higher spectral resolution. More abundant spectral information of ground features can detect more targeted spectral features. This is more conducive to the accurate identification of features. By combining the spatial information obtained by the imaging spectrometer, the hyperspectral image contains abundant one-dimensional spectral information and two-dimensional spatial information at the same time, and all information is integrated to form a three-dimensional data cube. Hyperspectral images can generally be represented by three methods: image space, spectral space, and feature space. The image space intuitively provides the relationship between the image pixel value and the spatial position of the ground object. The spectral space provides the relationship between the spectral curve of the pixels in the image and the category of ground objects. The feature space is a point that sets a pixel point as a d-dimensional space.

Image space is the traditional representation of remote sensing images. The image quality is related to the spatial resolution, because it can intuitively give the positional relationship between the radiation information of the ground object and the surrounding pixels. When the spatial resolution is good, the examiner can directly judge the pixel category through the image. Each pixel in the image space can find a corresponding spectral curve that varies with wavelength in the spectral space. The higher the spectral resolution, the more absorption or reflection features it contains that can identify the type of object. Each point in the feature space can be represented by a multidimensional vector, which reflects the position distribution and variety law of the ground features. In the task of hyperspectral classification, the statistical characteristics of a certain pixel and neighborhood in the image space can provide the spatial characteristics of the sample. Combined with spectral features as the input of the classifier, it can play a role in mentioning the classification accuracy.

In hyperspectral data, the band data increase sharply and the width decreases, which indicates that the spectral resolution of the data is very high. If the ground features are classified in detail, sufficient spectral information can be obtained in multiple spectral bands, but by densely arranging the bands, the adjacent bands can have a specific correlation and the hyperspectral data can have a certain degree of redundancy. From the statistical analysis point of view, because all bands correspond to the same spatial field of view, the analysis of the correlation between the bands is of great significance. This provides a theoretical basis for further processing of data and better use of spectral information.

Hyperspectral data can explain the subtle changes in ground features caused by its rich spectral bands, but it will also become more difficult to classify the same ground features scattered in different environments. Under normal circumstances, even for the same type of ground object, the performance of the spectral curve will be different due to different environments. In most cases, the spectral spatial distribution of different substances is also different. Therefore, it is difficult to distinguish features with similar spectral characteristics.

Hyperspectral remote sensing images provide rich spatial information and high-resolution spectral information. This provides more different types of features for image processing. For the same type of ground objects in the same scene, the spatial and spectral information contained may have great similarities. It essentially shows their identity. Fully considering the structural advantages of the integration of hyperspectral data and spectra, and realizing the joint utilization of space and spectral information, it extracts more targeted features and plays a synergistic effect between different types of features, to improve the feature extraction and classification of hyperspectral data.

## 3. Atlas Data Set Experiment

### 3.1. Hyperspectral Image Data Set Experiment

In this experiment, two data sets were selected: Indian Pines [24] and Pavia University [25]. Due to the high dimensionality of hyperspectral images and the greater redundancy between spectra, the PCA algorithm is first used to reduce the dimensionality of the network. After the experiments, when the principal component is selected as 30, the data dimensionality reduction can guarantee more than 99% of the information of the original data, so the principal component is selected as 30 to reduce the dimensionality of the two data sets first. The convolutional encoder is shown in Table 1.

Table 1 is the network architecture designed in this article for three-dimensional data samples. The network has 3 three-dimensional convolutional layers and two fully connected layers. A three-dimensional CNN is used to extract the local joint information of hyperspectral images in space and spectrum at the same time, and a fully connected neural network is used to perform a layer of nonlinear mapping, so that the global information of the sample can be finally obtained, and finally the classification is performed. In the three-dimensional convolutional network encoder, this article uses batch normalization for processing, which can make the model converge faster, speed up the training of the network, and enable the model to learn with a larger learning rate.

#### 3.1.1. Experimental Results and Analysis of Indian Pines Data Set

To prove the effectiveness of the algorithm, in this chapter, four hyperspectral image classification algorithms will be compared and analyzed. These are based on the support vector machine (SVM) method and the three-dimensional convolutional neural network method (3DCN). The 3D convolutional encoder network designed in this article is used for the fully supervised training method (BASE-3DCNN) and the residual neural network method based on space spectrum combination (SSRN). The input of these methods is set to the same 27*∗*27*∗*30 data block as the input size of this article. Among them, SVM, 3DCNN, BASE-3DCNN, and SSRN are all fully supervised classification methods. The Gaussian kernel used by SVM is a nonlinear classifier, and the input is expanded into a one-dimensional data block. 3DCNN is the first paper method that uses three-dimensional CNNs in the field of hyperspectral image classification. It uses the network structure and parameters in the original paper. BASE-3DCNN is the network structure of the encoder in the algorithm of this chapter combined with the empty spectrum of the semisupervised CNN (S4CNN), which can prove the effect of unlabeled samples on the classification results. SSRN is a model based on residual neural network, which can extract the information between space and spectrum at the same time and use the jump connection in the residual network to deepen the network while making the network still able to train. It can achieve the best effect in the current supervised classification.  Table 2 shows the comparison results of different methods.

The classification result is mainly divided into two parts: one is the ground feature distribution map obtained by classification, which can intuitively see the effect of classification; and the other is the accuracy of classification, this part directly compares the quality of classification with the numerical value.

Table 2 can prove the superiority of the algorithm in this chapter on this data set from the data: first, look at the three main criteria for judgment. From the OA, AA, and kappa coefficients, the algorithm in this chapter is the best among the five hyperspectral image classification algorithms. From the perspective of OA alone, the algorithm in this chapter is 9% higher than the SVM algorithm, 6.1% higher than the 3DCNN algorithm, 1.5% higher than the BASE-3DCNN algorithm, and 0.5% higher than the SSRN method. From the AA point of view, the algorithm in this chapter is 15.3% higher than the SVM algorithm, 13.2% higher than the 3DCNN algorithm, 2.3% higher than the BASE-3DCNN algorithm, and 7.2% higher than the SSRN method. From the kappa coefficient, the algorithm in this chapter is 10.3% higher than the SVM algorithm, 5.2% higher than the 3DCNN algorithm, 1.7% higher than the BASE-3DCNN algorithm, and 0.7% higher than the SSRN method.

#### 3.1.2. Experimental Results and Analysis of Pavia University Data Set

The comparison results of S4CNN in the Pavia University data set and different methods are shown in Table 3.

Table 3 from the observation of the data, the algorithm in this chapter is superior to several other algorithms: the algorithm in this chapter is the best among the five hyperspectral image classification algorithms. From the perspective of OA alone, the algorithm in this chapter is 4.7% higher than the SVM algorithm, 3.3% higher than the 3DCNN algorithm, 2.1% higher than the BASE-3DCNN algorithm, and 0.5% higher than the SSRN method. From the AA point of view, the algorithm in this chapter is 6.7% higher than the SVM algorithm, 5.2% higher than the 3DCNN algorithm, 2.4% higher than the BASE-3DCNN algorithm, and 0.3% higher than the SSRN algorithm. From the perspective of kappa coefficient, the algorithm in this chapter is 6.3% higher than the SVM algorithm, 4.4% higher than the 3DCNN algorithm, 2.6% higher than the BASE-3DCNN algorithm, and 0.7% higher than the SSRN method.

### 3.2. Feature Extraction Strategy Experiment

This article will carry out the experiment of feature extraction strategy in this part, as the feature extractor is the encoder after training, and it needs to send the feature extractor to the classifier if it wants to verify the effect of the feature. Tables 4 and 5 show the influence of different feature extraction strategies on the two data sets. In Tables 4 and 5, “un-pooling” means that no pooling operation is performed before the features are sent to the classifier, while “pooling” means that the features are first subjected to maximum pooling processing, and then sent to the classifier.

From Tables 4 and 5, it can be seen that after the pooling operation, the OA value of the first convolutional layer h1 of the encoder reaches 91.61%, which is 44.72% higher than the first layer feature without pooling operation. After the pooling operation, the best effect among the three layers of the encoder is the second layer h2, and the OA value is 93.81%. However, after connecting h1 and h2, the value of OA reaches 96.15%, which is higher than that of h1 and h2 alone. The experiment on the Salinas data set has the same conclusion. After pooling and combining the features of the first layer and the second layer, the largest OA value is obtained, which is 98.99%.

**Figure 7:**
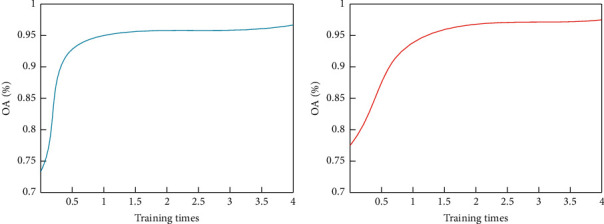
Classification results of 2D-SACNN on two data sets training: (a) Indian Pines data set and (b) Salinas data set.

The classification results of 2D-SACNN on different data sets during the training process are shown in Figure 7.

From Figure 7, it can be seen that in the early stage of training, the OA value of 2D-SACNN rises rapidly as the training progresses. However, in the following period, the classification accuracy rose slowly and finally converged to a maximum value, which means that the network finally obtained the optimal parameters. Specifically, for the Indian Pines data set and the Salinas data set, 2D-SACNN starts to converge after 3000 and 10000 iterations, respectively. After 40,000 iterations, the classification accuracy of 2D-SACNN on the Indian Pines data set and Salinas data set is 95.83% and 98.29%, respectively. Experiments show that as the training progresses, 2D-SACNN can converge to a maximum value, which proves the convergence of the method.

## 4. Diagnosis of Microscopic Hyperspectral Images of Hepatobiliary Tumors Based on Convolutional Neural Network

### 4.1. Super-Resolution Performance of SDCNN Model

**Figure 8:**
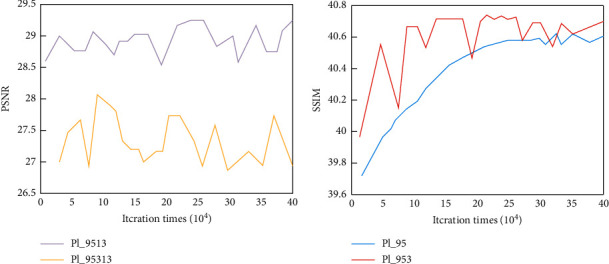
PSNR comparison chart of (a) four-layer SDCNN network and five-layer SDCNN network and (b) three-layer SDCNN network and two-layer SDCNN network.

In order to learn an SDCNN model that can fully characterize the mapping relationship, we first test the corresponding performance of SDCNN under different network layers, to select the best network layer. When the number of layers is determined, what needs to be further determined is the size of the convolution kernel of different layers. During the experiment, by measuring the average PSNR and SSIM values between the output spectral difference of SDCNN and the reference spectral difference, the performance comparison of SDCNN with different network structures is evaluated. The performance comparison of the three-layer and four-layer SDCNN network structure is shown in Figure 8.

It can be seen from Figure 8 that as the number of iterations increases, the PSNR and SSIM values of the three-layer SDCNN network structure first increase and then gradually become stable. The performance of the four-layer SDCNN network structure is unstable and is always worse than the performance of the three-layer network structure.

**Figure 9:**
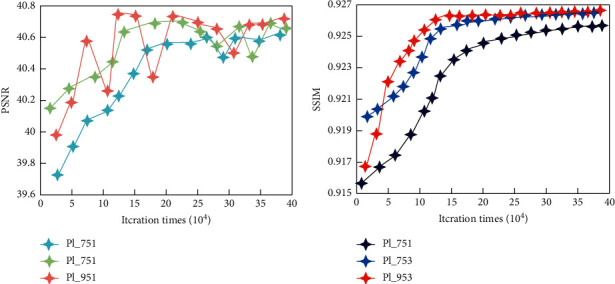
Comparison of (a) three groups of plPSNR with different convolution kernel sizes and (b) three groups of plSSIM with different convolution kernel sizes.

When the number of layers is determined, we need to find the best convolution kernel size for each layer. In the experiment, we refer to the architecture of the SDCNN network and design three different sets of convolution kernel parameters (9-5-3, 7-5-3, and 7-5-1). The comparison of the average PSNR and SSIM values of the three SDCNN networks with different convolution kernel sizes under the same number of iterations is shown in Figure 9.

It can be seen from Figure 9 that as the number of iterations increases, the overall trend in PSNR and SSIM of SDCNN networks with different convolution kernel sizes begins to increase and then gradually stabilizes. At the same time, it can be seen that the SDCNN with a convolution kernel size of 9-5-3 performs better than other models.

### 4.2. Diagnosis of Microscopic Hyperspectral Images of Hepatobiliary Tumors Based on Convolutional Neural Network

**Figure 10:**
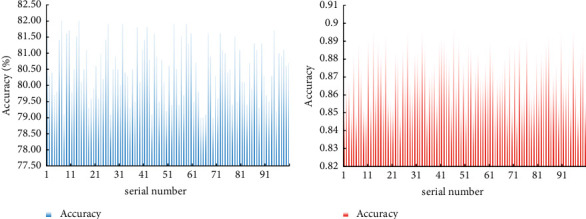
Experimental results of two diagnostic methods. (a) Accuracy of traditional diagnostic methods. (b) Accuracy of the method based on convolutional neural network.

To compare the accuracy of the diagnosis method for hepatobiliary tumors based on the microscopic hyperspectral image diagnosis of the CNN designed in this article and the traditional diagnosis methods, this article designs a set of comparative experiments. The specific steps of the experiment are as follows: two diagnostic methods were used to diagnose 100 groups of patients with hepatobiliary tumors, and the accuracy of the diagnosis results is shown in Figure 10.

It can be seen from Figure 10 that the accuracy of the diagnosis method based on the CNN is 85%–90%, while the accuracy of the traditional diagnosis method is only 79%–82%. This shows that the hepatobiliary tumor microscopic hyperspectral image diagnosis method based on the CNN can effectively improve the accuracy of the diagnosis of hepatobiliary tumors.

## 5. Conclusions

This article mainly studies the image diagnosis problem of the specular Atlas of liver and gallbladder tumors. Through the combined use of CNNs, machine learning is performed on it, which effectively improves the classification and accuracy of the specular Atlas. At the same time, this article designs a hyperspectral image data set experiment and a feature extraction strategy experiment. The experiment analyzes the influencing factors of the two data sets in the CNN. Then this article improves the performance of hyperspectral images through the SCDND model, and finally compares it with traditional diagnostic methods. The results show that the diagnostic accuracy of the microscopic hyperspectral image based on the CNN is as high as 85%–90%, which is an increase of 6%–8% compared with the traditional method, which is 79%–82%.

## Figures and Tables

**Table 1:** Convolution structure of 3D convolutional encoder.

Number of layers	Input size	Feature number	Number of convolution kernels	Convolution kernel size	Step size	Filling method	Pooling size	Whether batch normalization	Activation function
1	27*∗*27*∗*30	1	32	4*∗*4*∗*5	(1,1,1)	Valid	2*∗*2*∗*2	Yes	Relu
2	12*∗*12*∗*13	32	64	5*∗*5*∗*4	(1,1,1)	Valid	2*∗*2*∗*2	Yes	Relu
3	4*∗*4*∗*5	64	128	3*∗*3*∗*4	(1,1,1)	Valid	2*∗*2*∗*2	Yes	Relu
4	1	128	500	128	—	—	—	Yes	Relu
5	1	500	n	500	—	—	—	—	Softmax

**Table 2:** Comparison results of S4CNN with different methods in the Indian Pines data set.

Method	Type				
	SVM	3DCNN	BASE-3DCNN	SSRN	S4CNN
Corn-notill	87.33	83.46	69.55	30.44	100.00
Corn-min	80.13	92.28	97.54	98.49	95.41
Corn	52.31	88.13	87.96	97.89	97.43
Grass-pasture	91.30	87.18	95.43	92.51	99.58
Grass-trees	99.73	91.36	97.24	96.73	98.07
Grass-pasture-mowed	67.88	35.71	100.00	100.00	100.00
Hay-windrowed	100.00	100.00	100.00	100.00	100.00
OA (%)	88.70	93.15	96.15	97.12	97.71
AA (%)	81.56	83.63	94.52	89.64	96.87
Kappa × 100	87.06	92.16	95.62	96.72	97.39

**Table 3:** Comparison results of S4CNN with different methods in the Pavia University data set.

Method	Type				
	SVM	3DCNN	BASE-3DCNN	SSRN	S4CNN
Asphalt	94.21	92.31	97.78	98.41	98.68
Meadows	98.43	98.01	98.47	99.56	99.94
Gravel	75.31	84.86	94.73	92.48	97.66
Trees	92.11	97.02	92.25	95.34	98.04
Painted metal sheets	99.84	97.93	99.64	99.43	99.92
Bare soil	90.01	98.94	100.00	99.26	99.84
Shadows	93.67	96.44	96.14	97.82	97.88
OA (%)	94.39	95.76	96.97	98.57	99.11
AA (%)	91.64	93.09	95.93	98.03	98.32
Kappa × 100	92.53	94.39	96.21	98.12	98.82

**Table 4:** Experimental results of different feature extraction strategies on the Indian Pines data set.

	Un-pooling		Pooling	
	OA (%)	Feature size	OA (%)	Feature size
h1	46.89	4704	91.61	96
h2	23.97	4800	93.81	192
h3	23.97	3456	77.32	384
h1,h2	23.97	9504	96.15	288
h1,h3	23.97	8160	76.94	480
h2,h3	23.97	8256	74.83	576
h1,h2,h3	23.97	12960	74.12	672

**Table 5:** Experimental results of different feature extraction strategies on the Salinas data set.

	Un-pooling		Pooling	
	OA (%)	Feature size	OA (%)	Feature size
h1	94.38	4704	98.24	96
h2	94.00	4800	98.82	192
h3	93.70	3456	97.86	384
h1,h2	84.26	9504	98.99	288
h1,h3	94.17	8160	98.23	480
h2,h3	94.02	8256	98.46	576
h1,h2,h3	94.17	12960	98.60	672

## Data Availability

All the data used are given in the article.
